# SR Protein Kinases Regulate the Splicing of Cardiomyopathy-Relevant Genes via Phosphorylation of the RSRSP Stretch in RBM20

**DOI:** 10.3390/genes13091526

**Published:** 2022-08-25

**Authors:** Mingming Sun, Yutong Jin, Yanghai Zhang, Zachery R Gregorich, Jun Ren, Ying Ge, Wei Guo

**Affiliations:** 1 Department of Zoology and Physiology, University of Wyoming, Laramie, WY 82071, USA; 2 Department of Chemistry, University of Wisconsin-Madison, Madison, WI 53706, USA; 3 Department of Animal and Dairy Sciences, University of Wisconsin-Madison, Madison, WI 53706, USA; 4 Department of Cardiology, Shanghai Institute of Cardiovascular Diseases, Zhongshan Hospital Fudan University, Shanghai 200032, China; 5 Department of Cell and Regenerative Biology, School of Medicine and Public Health, University of Wisconsin-Madison, Madison, WI 53706, USA; 6 Human Proteomics Program, School of Medicine and Public Health, University of Wisconsin-Madison, Madison, WI 53705, USA

**Keywords:** RBM20, phosphorylation, SR protein kinase, mRNA splicing, cardiomyopathy

## Abstract

(1) Background: RNA binding motif 20 (RBM20) regulates mRNA splicing specifically in muscle tissues. Missense mutations in the arginine/serine (RS) domain of RBM20 lead to abnormal gene splicing and have been linked to severe dilated cardiomyopathy (DCM) in human patients and animal models. Interestingly, many of the reported DCM-linked missense mutations in RBM20 are in a highly conserved RSRSP stretch within the RS domain. Recently, it was found that the two Ser residues within this stretch are constitutively phosphorylated, yet the identity of the kinase(s) responsible for phosphorylating these residues, as well as the function of RSRSP phosphorylation, remains unknown. (2) Methods: The ability of three known SR protein kinases (SRPK1, CLK1, and AKT2) to phosphorylate the RBM20 RSRSP stretch and regulate target gene splicing was evaluated by using both in vitro and in vivo approaches. (3) Results: We found that all three kinases phosphorylated S638 and S640 in the RSRSP stretch and regulated RBM20 target gene splicing. While SRPK1 and CLK1 were both capable of directly phosphorylating the RS domain in RBM20, whether AKT2-mediated control of the RS domain phosphorylation is direct or indirect could not be determined. (4) Conclusions: Our results indicate that SR protein kinases regulate the splicing of a cardiomyopathy-relevant gene by modulating phosphorylation of the RSRSP stretch in RBM20. These findings suggest that SR protein kinases may be potential targets for the treatment of RBM20 cardiomyopathy.

## 1. Introduction

RNA binding motif 20 (RBM20) is a splicing factor that is highly expressed in heart muscles [1,2,3]. RBM20 is the major regulator of alternative splicing of the *Ttn* gene [1,2,4,5], which encodes the giant sarcomeric protein titin. Titin is a functionally pleiotropic protein that serves as a molecular blueprint for the maintenance of sarcomere integrity and force transduction [6,7], a molecular spring that defines muscle stiffness [8,9], and a molecular signaling mediator for muscle hypertrophy and protein quality control [10,11,12]. Beyond *Ttn*, RBM20 has been shown to regulate the splicing of over 30 genes, including the contractile gene myosin heavy chain 6 (*Myh6*), as well as calcium-handling genes such as ryanodine receptor 2 (*Ryr2*) and calcium/calmodulin-dependent protein kinase type II d (*Camk2d*) [2,5,13,14,15]. Genetic ablation of *Rbm20* results in aberrant gene splicing and dilated cardiomyopathy (DCM) in rodents [2,16]. Furthermore, recent studies have shown that missense mutations in the arginine/serine (RS) domain—particularly within a conserved RSRSP stretch—of RBM20 also lead to aberrant splicing and severe DCM in patients and animal models [17,18,19,20,21,22,23,24,25,26]. 

Like other SR family proteins, RBM20 contains an RNA recognition motif (RRM) located at the N-terminus and a C-terminal RS domain [27,28,29]. Both the RRM and RS domains play an essential role in defining the splicing sites and the selection of exon/intron boundaries [27,28,29,30]. Prior studies have shown that multiple serine residues in the RS domains of SR family proteins can be phosphorylated by kinases, including SR protein-specific kinases (SRPKs), cdc2-like kinases (CLKs), and protein kinase B (PKB or AKT) [31,32,33]. These kinases modulate key cellular signaling pathways such as the PI3K/Akt signaling pathway [31,32,33]. Both hyper- and hypo-phosphorylation of residues within the RS domain(s) of splicing factors can suppress splicing reactions [34] and promote shuttling between the nucleus and the cytoplasm [35,36]. Although SR protein phosphorylation is essential for assembly of the spliceosome [35,37], dephosphorylation has also been shown to be important for splicing catalysis, as well as the export of splicing factors and processed mRNA to the cytoplasm for protein translation [37,38,39]. Thus, careful control of the extent of RS domain phosphorylation in SR proteins is essential for numerous steps in splicing control.

An earlier study demonstrated that two Ser residues within the RSRSP stretch in RBM20 are constitutively phosphorylated [40]. Recently, our group confirmed phosphorylation of these residues (S638 and S640) and identified additional sites of phosphorylation in and outside the RBM20 RS domain by using middle-down mass spectrometry (MS) [26]. However, the kinases responsible for phosphorylating the RSRSP stretch in RBM20 remain to be identified. In the present study, we sought to determine whether the kinases AKT2, CLK1, and SRPK1, which have previously been shown to phosphorylate the RS domain in other splicing factors, can phosphorylate S638 and S640 in the RBM20 RSRSP stretch. Using a combination of co-transfection experiments in cell culture, in vitro kinase assays, and investigation of RBM20 phosphorylation and target gene splicing in gene-edited animals, we confirmed that SRPK1, CLK1, and AKT2 interact with and regulate RBM20 phosphorylation and target splicing both in vitro and in vivo.

## 2. Materials and Methods

### 2.1. Experimental Animals and Sample Preparation

This study was performed with rat crosses of Sprague-Dawley (SD) × Brown Norway (BN) (all strains were originally obtained from Harlan Sprague Dawley, Indianapolis, IN, USA). Animals were maintained on standard rodent chow. This study was carried out in strict accordance with the recommendations in the Guide for the Care and Use of Laboratory Animals published by the National Institutes of Health. All procedures were approved by the Institutional Animal Care and Use Committee of the University of Wyoming. Primary neonatal cardiomyocytes were isolated and cultured from one-day-old rats. Mouse heart tissues from constitutively activated AKT mice mimicking the overexpression of AKT (overexpression mouse model, 2-month-old) and AKT2 knockout (KO) mice (2-month-old) were used in this study [41]. All samples for protein gel electrophoresis were prepared as described previously [2,42].

### 2.2. Neonatal Rat Cardiomyocyte (NRCMs) Isolation, Culture, and Treatment 

NRCMs were isolated from one-day-old rats, using the neonatal cardiomyocyte isolation system, as described previously [43,44]. The cells were re-suspended in complete media (M199/DMEM media supplemented with 20% fetal calf serum (FCS) and 1% penicillin/streptomycin), plated at a density of 1 × 10^6^ cells per cm^2^, and maintained in 5% CO_2_ at 37 °C. Cells were cultured for two days in complete media; supplemented with either the SRPK1 inhibitor SRPIN340 (25 μM) (Sigma-Aldrich, St. Louis, MO, USA, cat#SML1088) or the CLK1 inhibitor TG003 (10 μM) (Sigma-Aldrich, cat#T5575); and collected after 0 min, 5 min, 1 h, 6 h, 12 h, 24 h, or 48 h. Treatment with vehicle (DMSO) was used as a control. Harvested cells were lysed for protein and RNA preparation. 

### 2.3. Plasmid Constructs

Plasmids of pEGFP-C1-8his-RBM20 WT/mutations, pcDNA3.1-mcherry-SRPK1, and pcDNA3.1-mcherry-CLK1 were constructed by Gene Universal (Newark, DE, USA); pcDNA3-Myr-HA-Akt2 was a gift from William Sellers (Addgene plasmid # 9016); and pcDNA3.1-E64-70 expressing titin exons 64–70 was constructed as described previously [5].

### 2.4. RT-PCR Analysis 

HeLa cells were grown in DMEM media supplemented with 10% FBS. The titin minigene exon 64–70 construct was co-transfected, along with either WT or mutant *Rbm20* constructs, respectively, in HeLa cells, using Lipofectamine 2000 (Invitrogen, Carlsbad, CA, USA) according to the manufacturer’s instructions. Cells were harvested for protein and RNA preparation at 48 h post-co-transfection. RT-PCR was performed to detect exon inclusion or exclusion, using the forward and reverse primers 5′-ACCAGCTGTGCACACAAAGA-3′ and 5′-TCTTCTTTGCCACAGGAACG-3′, respectively. Endogenous titin splicing in NRCMs treated with different inhibitors was assessed by using the forward and reverse primers 5′-TGCCAAAGGTAGTGATCTCCG-3′ and 5′-GTGGTCTGCTGAGCATAGGAT-3′, respectively. The results were confirmed in three independent experiments, and the PCR products were analyzed on ethidium bromide agarose gels, as described previously [5]. 

### 2.5. Protein Expression and Purification from BL21(DE3)

Purified RBM20 was purchased from General Biosystems (Durham, NC, USA). RBM20 was cloned into the pET-22b vector, with the inclusion of the 8xHis-tag at the C-terminus. This plasmid was transformed into BL21(DE3) competent cells, and the expression was induced by the addition of IPTG (final concentration: 0.5 mM). Following induction, bacteria were cultured at 37 °C, pelleted, and lysed, and His-tagged RBM20 was purified by affinity purification, using an anti-His-tag antibody.

### 2.6. In Vitro Kinase Assays

In vitro profiling of the kinase panel was performed at Reaction Biology Corporation, using the “HotSpot” assay platform. Briefly, 5 μM purified RBM20 protein [24,26] was incubated with 10 nM AKT2, 12.5 nM CLK1, 0.8 nM SRPK1, 1.5 nM CLK2, or 0.1 nM SRPK2 in reaction buffer (20 mM HEPES pH 7.5, 10 mM MgCl_2_, 1 mM EGTA, 0.02% Brij35, 0.02 mg/mL BSA, 0.1 mM Na_3_VO_4_, 2 mM DTT, and 1% DMSO). To assess the activity of different kinases, the same amount of AKT2, CLK1, SRPK1, CLK2, or SRPK2 was incubated with or without a standard substrate (crosstide for AKT2, MBP for CLK1 and CLK2, and RS peptide for SRPK1 and SRPK2) in the same reaction buffer. Compounds were delivered into the reaction, followed 20 min later by the addition of a mixture of non-radioactive ATP (Sigma-Aldrich) and γ-^33^P ATP (PerkinElmer, Waltham, MA, USA) to a final concentration of 10 μM. Reactions were carried out at 25 °C for 120 min, followed by spotting of the reactions onto P81 ion exchange filter paper (Sigma-Aldrich). Unbound phosphate was removed by extensive washing of filters in 0.75% phosphoric acid. The phosphorylated signal for each reaction was calculated in nM [45]. 

### 2.7. Middle-Down Mass Spectrometry (MS)

Following in vitro kinase assays, RBM20 phosphorylation was assessed by using middle-down MS as previously described [26]. Briefly, RBM20 was digested and subjected to online liquid chromatography (LC)–MS and tandem MS (MS/MS) analyses [46,47], as well as offline high-resolution MS/MS analysis of phosphorylated peptides [48]. The online LC–MS and MS/MS data were processed and analyzed by using DataAnalysis software from Bruker Daltonics (Billerica, MA, USA). Mass spectra were deconvoluted by using the Maximum Entropy algorithm in the DataAnalysis software. For online MS/MS data, the output from the DataAnalysis software was analyzed by using MS-Align^+^ [48,49] to identify RBM20 peptides. The cutoff of E- and *p*-values was set to E-10 to ensure the confident identification of peptides. For offline MS/MS data, the mass and charge lists for fragment ions were output from the DataAnalysis software for peptide sequence identification, using MS-Align+. In-house developed MASH Suite Pro [50,51] was used for the manual validation of fragment ion assignment and the localization of phosphorylation sites. A minimum fit of 60% and S/N threshold of 3 were set for peak picking. Fragment ions, including c, c^−1^, z^●^, and z^●+1^ ions, were validated within 10 ppm mass error. All reported masses are the monoisotopic masses. 

### 2.8. Western Blot

Western blot analysis was performed as previously described [43,44]. Briefly, proteins extracted from cells or tissues were separated on SDS–PAGE gels and transferred to PVDF membranes. The membranes were probed with antibodies against AKT2 (Santa Cruz, CA, USA, cat#sc-81436), CLK1 (Santa Cruz, cat#sc-515897), SRPK1 (Santa Cruz, cat#sc-100443), pRBM20 (gift from Dr. Hidehito Kuroyanagi), HA-tag (Abcam, Cambridge, UK, cat#ab137838), His-tag (Abcam, cat#ab18184), and GAPDH (Cell Signaling, MA, USA, cat#14C10), which served as a protein loading control. 

### 2.9. Co-Immunoprecipitation (co-IP)

Co-IPs were carried out by using a Pierce Co-IP kit (Pierce, IL, USA), as per the manufacturer’s instructions. Briefly, 100 µg of purified antibodies was coupled with resin. Protein samples (1 mg) were incubated with the antibody-coupled resin for 2 h. Protein–antibody complexes were eluted in 50 µL elution buffer after mixing and washing. The eluted protein samples were subjected to immunoblotting [52].

### 2.10. Data Analysis

GraphPad Prism software was used for statistical analysis. Results are presented as mean ± SEM. Statistical significance for each variable was estimated by the paired t-test (two-tailed). Significance was considered as probability values of *p* < 0.05 indicated by one asterisk, *p* < 0.01 indicated by two asterisks, and *p* < 0.001 indicated by three asterisks. 

## 3. Results

### 3.1. Co-Transfection with AKT2, CLK1, or SRPK1 Increases Phosphorylation of the RBM20 RSRSP Stretch in HeLa Cells

**Figure 1:**
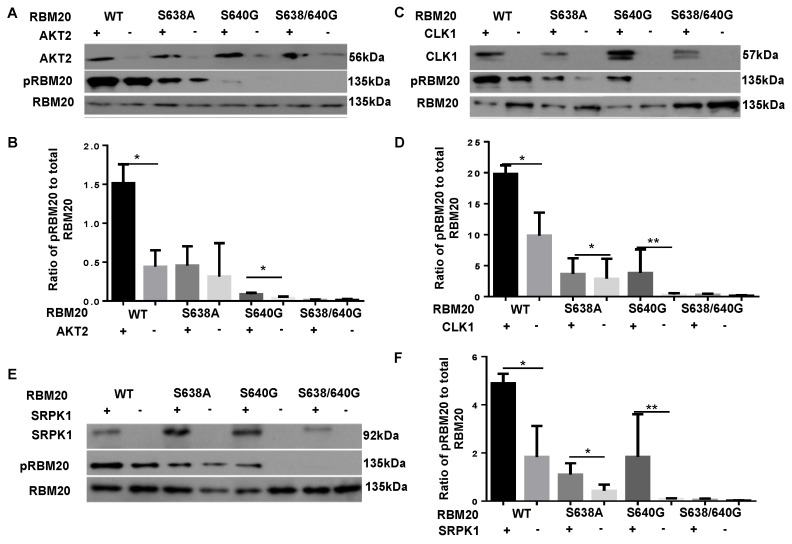
SR protein kinases phosphorylate serine residues in the RBM20 RSRSP stretch in HeLa cells (**A**,**C**,**E**). HeLa cells were co-transfected with plasmids carrying the SR protein kinases AKT2 (**A**), CLK1 (**C**), or SRPK1 (**E**) and WT or mutant RBM20. Cell lysates were subject to immunoblotting with antibodies against pRBM20, RBM20, AKT2, CLK1, and SRPK1. Blots shown are representative of three independent experiments. Transfection of SR protein kinase plasmids increased the expression of the respective kinases significantly (**B**,**D**,**F**). Quantification of RBM20 phosphorylation in HeLa cells co-transfected with individual kinases or empty plasmid (control). Data are shown as mean  ±  SD (*n* = 3); * *p* < 0.05, ** *p* < 0.01. WT, wild type.

Kinases belonging to the AKT, CLK, and SRPK kinase families have previously been shown to phosphorylate the RS domain in splicing factors [31,32,33]. To test whether kinases belonging to these families can also phosphorylate the RSRSP stretch in the RS domain of RBM20, plasmids carrying AKT2, CLK1, and SRPK1 were co-transfected with either WT or mutant RBM20 constructs in HeLa cells. HeLa cells were chosen because these cells do not express RBM20, thus allowing for the avoidance of confounding signals from endogenous protein. Co-transfection of WT or mutant RBM20 with empty plasmid (no kinase) was used as a control. Cells were harvested, and lysates were prepared 48 h post-co-transfection. Proteins from each treatment were subjected to Western blot analysis, using anti-phospho-RBM20 (gift from Dr. Hidehito Kuroyanagi) [40], which recognizes phosphorylation of the RSRSP stretch located in the RS domain of RBM20, and anti-pan-RBM20 (home-made) antibodies [2]. The Western blot analysis confirmed the increased expression of AKT2, CLK1, and SRPK1 in HeLa cells transfected with these kinases relative to cells transfected with empty plasmid (Figure 1A,C,E). Phosphorylation of the RSRSP stretch in WT RBM20 was significantly increased by co-transfection with all three kinases when compared to that in cells co-transfected with empty plasmid (Figure 1). Furthermore, co-transfection with each of the three kinases increased phosphorylation of the mutated RSRSP stretch in S638A and S640G RBM20, with the exception that phosphorylation of S638A RBM20 did not increase significantly in response to co-transfection with AKT2 (Figure 1). As expected, phosphorylation of the double mutant (S638A/S640G), which lacks the two phosphorylatable Ser residues in the RSRSP stretch, was not detected (Figure 1). Collectively, these data demonstrate that co-transfection with AKT2, CLK1, or SRPK1 increases the phosphorylation of the RSRSP stretch in RBM20-transfected HeLa cells.

### 3.2. CLK1 and SRPK1 Directly Phosphorylate the RBM20 RS Domain In Vitro 

**Figure 2:**
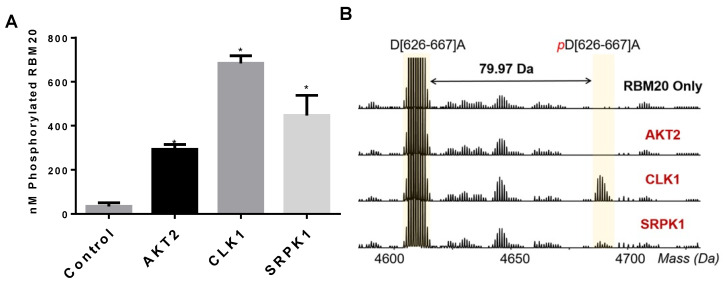
In vitro phosphorylation of RBM20 by AKT2, CLK1, and SRPK1. (**A**) In vitro kinase assay with individual SR protein kinases. Purified RBM20 (5 μM) was incubated with different kinases in kinase buffer containing 10 μM ^33^P-γ-ATP. The extent of ^33^P incorporation upon incubation with each individual kinase was determined in nM. (**B**) Middle-down LC–MS analysis with samples from the in vitro kinase assay. The main peak is 18× zoomed. Data are presented as mean ± SD (*n* = 3); * *p* < 0.05.

While increased phosphorylation of RBM20 upon co-transfection with AKT2, CLK1, or SRPK1 in HeLa cells suggests a role for these kinases in the regulation of RBM20 phosphorylation, it is possible that the mode of regulation is indirect. Next, to determine whether AKT2, CLK1, and SRPK1 can directly phosphorylate RBM20, we performed in vitro kinase assays with γ-^33^P-ATP and purified RBM20 (Figure S2A). An analysis of ^33^P incorporation confirmed that all three kinases phosphorylated RBM20 in vitro, with the lowest level of ^33^P incorporation detected for RBM20 incubated with AKT2 (Figure 2A and Figure S2B). Although this result shows that all three kinases can directly phosphorylate RBM20 in vitro, whether these kinases directly phosphorylate Ser residues within the RS domain could not be determined with this assay. To gain insight, phosphorylation of the RBM20 RS domain by the aforementioned kinases was assessed by using middle-down MS (Figure 2B and Supplementary Figure S1). The MS analysis confirmed the phosphorylation of the peptide encompassing the RBM20 RS domain by CLK1 and SRPK1, but not AKT2 (Figure 2B). Given that RBM20 phosphorylation by AKT2 was detected based on ^33^P incorporation, this result may indicate that the AKT2-mediated phosphorylation of Ser residues within the RBM20 RS domain has low efficiency in vitro or, alternatively, that regulation of the RBM20 RS domain phosphorylation by AKT2 is indirect. Taken together, these results show that CLK1 and SRPK1 can directly phosphorylate the RBM20 RS domain in vitro. On the other hand, while AKT2 can directly phosphorylate other sites in RBM20, it appears that this kinase does not directly regulate the phosphorylation of Ser residues within the RBM20 RS domain, at least in vitro.

### 3.3. AKT2, CLK1, and SRPK1 Interact with RBM20 in Co-Transfected HeLa Cells and Regulate Titin Pre-mRNA Splicing 

**Figure 3:**
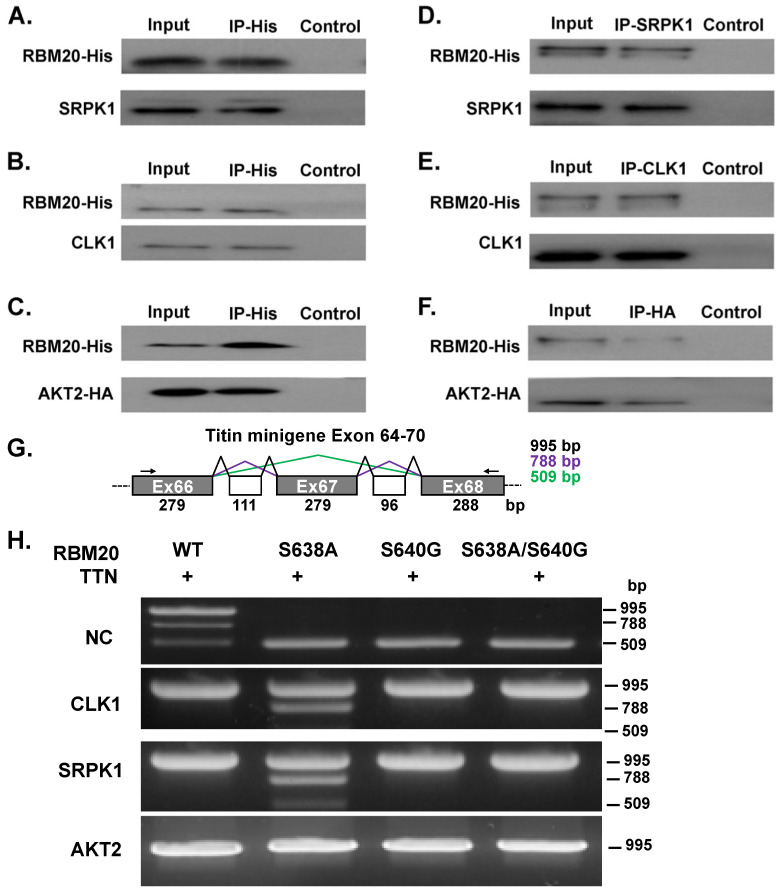
SR protein kinases interact with RBM20 and regulate *Ttn* pre-mRNA splicing in HeLa cells. (**A**–**C**) Pulldown of kinases with His-tagged RBM20 was assessed by using Western blot. (**D**–**F**) Pulldown of His-tagged RBM20 by SRPK1, CLK1, or HA-tagged AKT was evaluated by Western blot. (**G**). Schematic showing *Ttn* minigene splice variants with sizes indicated. (**H**). RT-PCR detection of *Ttn* minigene splice variants after co-transfection with individual SR protein kinases and either WT or mutant RBM20. Input, total protein before co-IP; IP–His, elution from co-IP with anti-His-tag antibody; Control, elution from co-IP without antibody conjugation to the beads; NC, control without kinase transfection; WT, RBM20 wild type.

Next, we sought to determine whether RBM20 interacts with AKT2, CLK1, and SRPK1 in co-transfected HeLa cells, using co-immunoprecipitation (co-IP). Plasmid containing RBM20 with an 8xHis-tag was co-transfected with individual kinase constructs in HeLa cells. Cells were harvested 48 h after co-transfection, and protein lysates were prepared and subjected to co-IP. An anti-His-tag antibody was used to capture RBM20 protein complexes, which were subsequently analyzed by Western blot, using anti-RBM20, anti-SRPK1, anti-CLK1, and anti-HA-tag antibodies. The Western blot analysis revealed that SRPK1 (Figure 3A), CLK1 (Figure 3B), and AKT2 (Figure 3C) immunoprecipitated with RBM20. In the reciprocal experiments, SRPK1, CLK1, and HA-tagged AKT2 were used as bait to capture RBM20. Consistently, the IP of all three kinases also pulled down RBM20 (Figure 3D–F). Notably, controls lacking antibody conjugated to the beads failed to capture any of the target proteins (Figure 3A–F). 

To determine whether these three kinases can also regulate titin pre-mRNA splicing, WT or mutant RBM20 was co-transfected along with a titin minigene exon 64–70 construct and individual kinase constructs in HeLa cells. A schematic showing titin minigene splice variants is displayed in Figure 3G, with the size of each variant indicated. Cells were harvested 48 h after co-transfection, total RNA was isolated, and RT-PCR was performed to detect changes in the splicing of the titin minigene construct. Three splice variants were detected in HeLa cells transfected with WT RBM20 in the absence of kinases (NC), while only the largest variant was detected in cells co-transfected with individual kinases (Figure 3H). In HeLa cells transfected with S638A-mutated RBM20 in the absence of kinases, only the smallest variant was expressed, whereas co-transfection with CLK1 or SRPK1, but not AKT2, shifted the expression pattern in such a way that expression of the two largest variants was favored; however, the smallest variant was still present, albeit at lower levels (Figure 3H). Unlike in HeLa cells transfected with S638A RBM20, co-transfection of S640G or double S638A/S640G mutant RBM20 with CLK1, SRPK1, or AKT2 promoted expression of only the largest splice variant (Figure 3H). These results demonstrate that CLK1, SRPK1, and AKT2 not only interact with RBM20 but can also regulate splicing of the RBM20 target gene, *Ttn*, in vitro.

### 3.4. Inhibition of CLK and SRPK Family Kinases Reduces RBM20 RSRSP Phosphorylation and Leads to Aberrant Pre-mRNA Splicing in NRCMs

**Figure 4:**
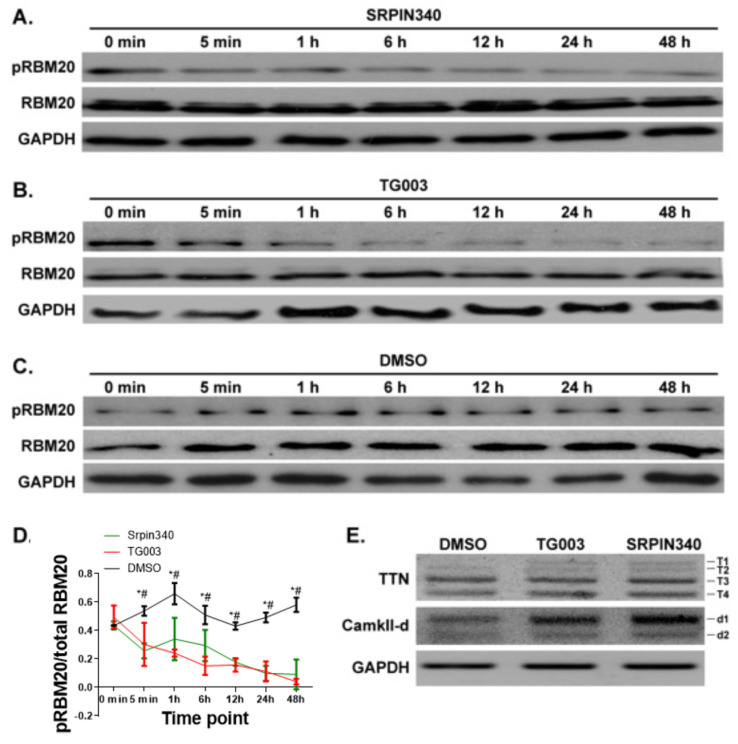
Impact of the inhibition of SRPK1 and CLK1 on RBM20 RSRSP stretch phosphorylation and target pre-mRNA splicing in vivo. (**A**–**C**) Western blot analysis of phospho- and pan-RBM20 in NRCMs treated with vehicle (DMSO), SRPIN340, or TG003 at different time points. (**D**) Quantification of Western blot results from (**A**–**C**). (**E**) RT-PCR analysis of *Ttn* and *Camk2d* splice variant expression in vehicle- or inhibitor-treated NRCMs. *Ttn* variants, T1–T4; *Camk2d* variants, d1 and d2. GAPDH served as a loading control for Western blot and housekeeping gene for normalization of RT-PCR data. Graph shows mean  ±  SD (*n* = 3); * *p* < 0.05 compared between SRPIN340 and DMSO, and # *p* < 0.05 compared between TG003 and DMSO.

Next, we tested whether the inhibition of endogenous SRPK and CLK family kinases impacts RBM20 RSRSP phosphorylation in isolated NRCMs. NRCMs were isolated from 1-day-old rats and treated with either SRPIN340 or TG003—selective inhibitors of SRPK and CLK kinases, respectively. NRCMs were harvested at different time points (0 min, 5 min, 1 h, 6 h, 12 h, 24 h, or 48 h) following inhibitor treatment, and RBM20 phosphorylation was assessed by Western blot. In agreement with the results of our previous experiments, we detected a time-dependent decrease in the phosphorylation of the RSRSP stretch in RBM20 beginning 5 min after the initiation of inhibitor treatment (Figure 4A,B,D). Conversely, phosphorylation of the RBM20 RSRSP stretch was not decreased following vehicle (DMSO) treatment (Figure 4C,D). Ttn and Camk2d are well-established targets of RBM20 [3]. Inhibition of kinases SRPK1 and CLK1 increased the expression of larger variants of both Ttn (t-1) and Camk2d (d-1) when compared to the control DMSO treatment (Figure 4E). Collectively, these results are in accordance with those of our previous experiments and verify that CLK and SRPK family kinases regulate phosphorylation of the RSRSP stretch within the RS domain of RBM20 and target splicing in vivo.

### 3.5. Phosphorylation of the RSRSP Stretch in RBM20 and Pre-mRNA Splicing of RBM20 Targets Are Altered in Akt2 KO and Transgenic Mice

**Figure 5:**
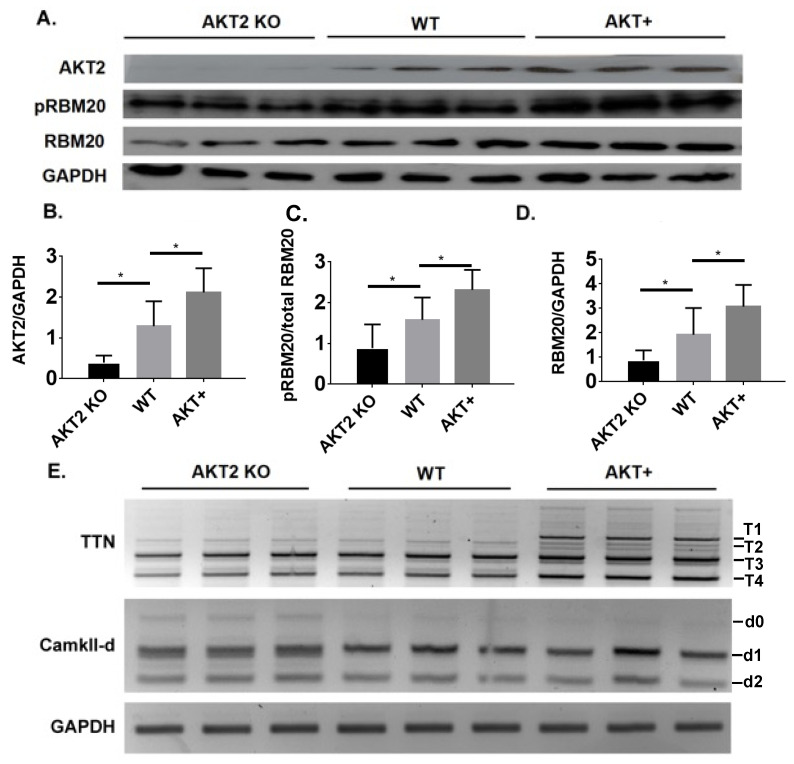
RBM20 phosphorylation and target splicing alterations in *Akt2* KO and overexpressing mice. (**A**) Western blot analysis of RBM20 phosphorylation, RBM20 expression, and AKT2 expression in *Akt2* KO and overexpressing mice. WT mice were used as control. (**B**) Quantification of AKT2 expression. (**C**) Quantification of RBM20 phosphorylation. (**D**) Quantification of RBM20 expression. (**E**) Detection of alterations in the splicing of *Ttn* and *Camk2d* pre-mRNAs, using RT-PCR, in the hearts of *Akt2* KO and overexpressing mice relative to WT. KO, knockout; WT, wild type; AKT+, AKT overexpression. T1–T4, *Ttn* splice variants; d1–d4, *Camk2d* splice variants; GAPDH served as a loading control for Western blot and housekeeping gene for normalization of RT-PCR data. Data are presented as mean ± SD (*n* = 3); * *p* < 0.05.

Due to the lack of specific AKT2 inhibitors, we evaluated the role of AKT2 in RBM20 phosphorylation in *Akt2* KO and overexpressing mice (Figure 5A,B). Heart tissues collected from WT mice were used as a control. The Western blot analysis revealed that RBM20 phosphorylation was significantly reduced and increased in the hearts of *Akt2* KO mice and *Akt2* overexpressing mice, respectively, compared to WT (Figure 5A,C). Consistent with our previous finding that AKT regulates RBM20 expression via modulation of the PI3K/AKT/mTOR signaling pathway [31,32], the expression of RBM20 was reduced and increased in the hearts of *Akt2* KO and overexpressing transgenic mice, respectively (Figure 5A,D). The splicing of RBM20 target transcripts was also altered in the hearts of these mice (Figure 5E). Specifically, the abundance of the larger d0 variant of Camk2d was increased in the hearts of *Akt2* KO mice, while the abundance of *Ttn* variants was unchanged (Figure 5E). Conversely, in the hearts of *Akt2* overexpressing mice, the abundance of larger *Ttn* transcript variants (T1 and T2) was increased, whereas the Camk2d variant abundances did not differ (Figure 5E). Collectively, these results provide additional evidence supporting a role for AKT2 in regulating phosphorylation of the RBM20 RSRSP stretch, either directly or indirectly, in vivo. These data also show that AKT2 regulates the splicing of RBM20 targets in the heart; however, whether this occurs through modulation of RBM20 phosphorylation, altered RBM20 expression, or both remains to be determined. 

### 3.6. Overexpression of CLK1, SRPK1, or AKT2 Alone Does Not Facilitate RBM20 Nucleocytoplasmic Transport

**Figure 6:**
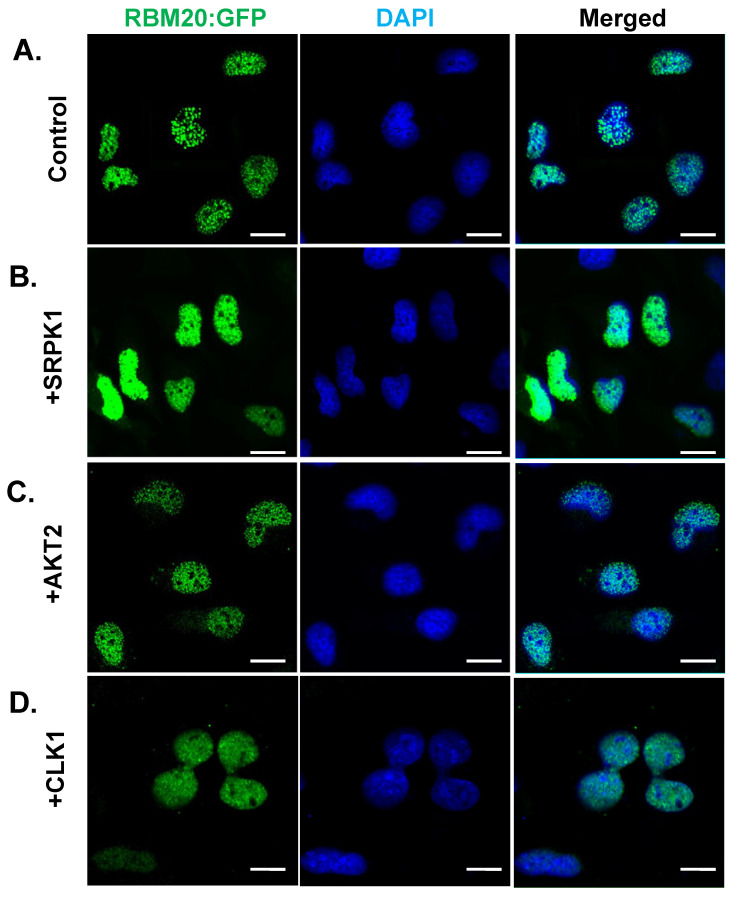
RBM20 localization in HeLa cells overexpressing CLK1, SRPK1, and AKT2. (**A**) Immunocytochemical staining of RBM20 in cells co-transfected with empty plasmid (no kinases control). (**B**–**D**) Immunocytochemical staining of RBM20 in cells co-transfected with SRPK1 (**B**), AKT2 (**C**), or CLK1 (**D**). Scale bars are 20 μm.

It is well-established that certain SR proteins (e.g., SRSF1) move continuously between the nucleus and the cytoplasm, and that this shuttling is facilitated by hyperphosphorylation [53]. Since overexpression of CLK1, SRPK1, or AKT2 increases RBM20 RSRSP stretch phosphorylation in co-transfected HeLa cells (Figure 1), we sought to determine whether increased phosphorylation of RBM20 promotes nucleocytoplasmic transport. HeLa cells were co-transfected with WT RBM20 and either CLK1, SRPK1, or AKT2, and the localization of RBM20 was assessed by immunocytochemistry 48 h post-co-transfection. Co-transfection of WT RBM20 with empty plasmid (no kinase) served as a control. As expected, RBM20 was localized to the nucleus of transfected HeLa cells without kinase overexpression. Interestingly, overexpression of CLK1, SRPK1, or AKT2 did not alter RBM20 localization (Figure 6A–D), suggesting that hyperphosphorylation of RBM20 phosphorylation alone does not promote shuttling between the nucleus and cytoplasm. 

## 4. Discussion

Herein, we demonstrated that phosphorylation of S638 (S635 in human) and S640 (S637 in human) in the RSRSP stretch of RBM20 is regulated by SRPK and CLK family kinases, as well as by AKT2. Specifically, we showed that these kinases interacted with and phosphorylated RBM20, using a combination of co-transfection experiments in HeLa cells and in vitro kinase assays. Moreover, we showed that CLK1 and SRPK1 directly phosphorylated the RS domain in RBM20, and the inhibition of endogenous CLK and SRPK family kinases in NRCMs confirmed such regulation of RSRSP stretch phosphorylation in the heart. Conversely, altered RBM20 phosphorylation in *Akt2* KO and overexpressing mice provided additional support for AKT2-mediated regulation of RBM20 RSRSP stretch phosphorylation in vivo; however, it remains unclear whether this regulation is direct or indirect. We also observed that SRPK1, CLK1, and AKT2 can regulate the pre-mRNA splicing of RBM20 target genes, which likely occurs via the modulation of RBM20 phosphorylation. 

Alternative pre-mRNA splicing is a common phenomenon in higher eukaryotes that increases protein diversity and serves as an additional regulatory mechanism governing gene expression in different cell types and during development [32]. The SR protein family of splicing factors and SR protein kinases play important roles in alternative pre-mRNA splicing in different ways, such as through spliceosome assembly, splicing catalysis, and signaling transduction [32]. The RS domain in SR proteins mediates a protein-protein interaction network to facilitate cross-intron interactions, exon definition, splice site selection, and the eventual formation of the higher-order spliceosome [54,55]. Posttranslational modifications are critical regulators of the protein–protein interactions mediated by the RS domain(s) of SR proteins [56]. At least three well-known posttranslational modifications occur on SR proteins: methylation, acetylation, and phosphorylation [57,58,59,60]. Currently, the most well understood of these modifications is phosphorylation, which is regulated by multiple kinases belonging to the CMGC family of kinases [61], as well as AKT [62,63]. The regulation of SR protein phosphorylation in vivo by three families of kinases, namely AKTs, SRPKs, and CLKs, has been confirmed through either genetic ablation or chemical inhibition [63,64,65,66,67,68]. In this study, we determined whether members of each of these kinase families can also regulate the phosphorylation of RBM20. We showed that AKT2, SRPK1, and CLK1 regulate RBM20 phosphorylation both in vitro and in vivo. Furthermore, we provided evidence that these kinases can modulate titin pre-mRNA splicing, thus making them potential therapeutic targets for the treatment of diastolic dysfunction [69]. 

SR protein phosphorylation plays a myriad of roles in the splicing of target transcripts. Prior studies have shown that phosphorylation is required for spliceosome assembly, but that dephosphorylation is crucial for splicing catalysis [70,71,72,73]. A specific phosphorylation state in some SR proteins is important to function properly in splicing, and partial, rather than full, phosphorylation of certain SR proteins is required for splicing activity [74,75]. Whether RBM20 phosphorylation plays a role in assembly of the spliceosome or splicing catalysis will need to be investigated in future studies. Aside from splicing, SR protein phosphorylation is also important for nuclear import and nucleocytoplasmic shuttling [36,53,76]. Based on the data presented herein, it appears that increased phosphorylation of RBM20 through kinase overexpression does not promote nucleocytoplasmic transport of the protein as it does for the shuttling SR protein SRSF1 [53]. This finding is consistent with our recently published data showing that constitutive pseudo-phosphorylation of phosphorylation sites within the RS domain in RBM20 by Ser-to-Asp mutagenesis does not promote nucleocytoplasmic transport of the protein in H9c2 cells [26]. Nevertheless, a notable caveat is that there is evidence in the literature suggesting that SR protein shuttling is also dependent on RNA binding and HeLa and H9c2 cells to not express titin, the primary target of RBM20. Thus, confirmation of these results in cells that express titin (e.g., cardiomyocytes) is warranted. Additional studies will also be necessary to determine the role of RBM20 RS domain phosphorylation in nuclear import. Taken together, our results provide new information on RBM20 phosphorylation. Further studies deciphering the role of RBM20 phosphorylation in alternative splicing in muscle tissues may aid the development of new strategies for the treatment of cardiomyopathies.

## Data Availability

Not applicable.

## Supplementary Materials

The following supporting information can be downloaded at: https://www.mdpi.com/article/10.3390/genes13091526/s1, Figure S1: LC-MS/MS analysis of samples from the in vitro kinase assay showing the presence of phosphorylation in peptide D[626-667]A; Figure S2: Coomassie staining for purified RBM20 and kinase activity assay with RBC substrates.
