# Supplementary material for: SR Protein Kinases Regulate the Splicing of Cardiomyopathy-Relevant Genes via Phosphorylation of the RSRSP Stretch in RBM20
Source: Genes (Basel). 2022 Aug 25;13(9):1526. doi: 10.3390/genes13091526 (PMC9498672; doi:10.3390/genes13091526)
Supplement: Supplementary file 1 [file genes-13-01526-s001.zip › genes-1828072-supplementary.pdf]

Supplement materials

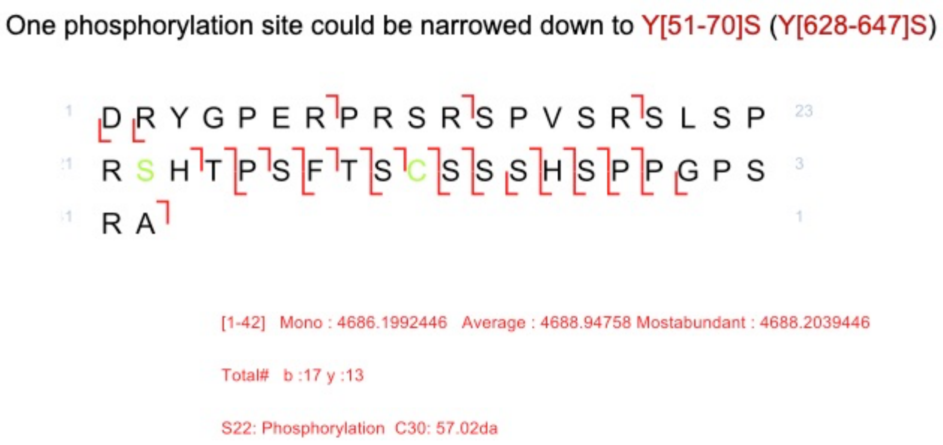

Figure S1. LC-MS/MS analysis of samples from the in vitro kinase assay showing the presence of phosphorylation in peptide D[626-667]A.

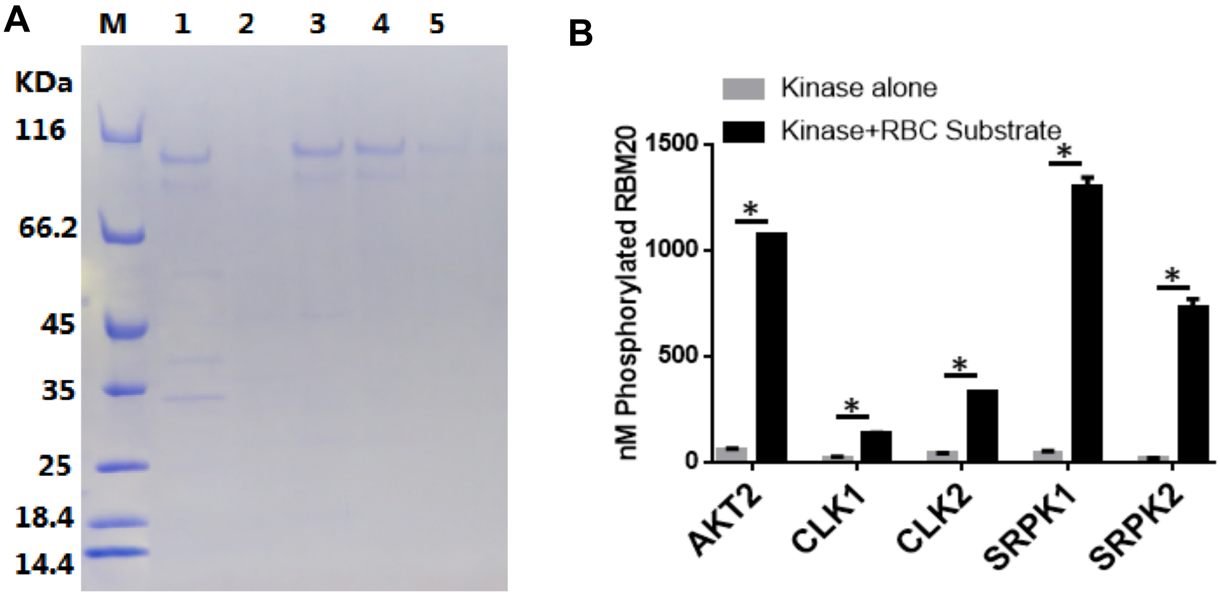

Figure S2. Coomassie staining for purified RBM20 and kinase activity assay with RBC substrates. A. Lane M: Protein Marker; Lane 1: Supernatant; Lane 2: Flow through; Lane 3: Wash 1 Lane 4: Wash 2; Lane 5: Elution；B. In vitro kinase assay with individual SR protein kinases and Reaction Biology Corporation (RBC) standard substrate, mean ± SEM (n=2) * $P < 0.05$.
